# Complete Genome Sequence of a Genotype XVII Newcastle Disease Virus, Isolated from an Apparently Healthy Domestic Duck in Nigeria

**DOI:** 10.1128/genomeA.01716-15

**Published:** 2016-02-04

**Authors:** Ismaila Shittu, Poonam Sharma, Tony M. Joannis, Jeremy D. Volkening, Georgina N. Odaibo, David O. Olaleye, Dawn Williams-Coplin, Ponman Solomon, Celia Abolnik, Patti J. Miller, Kiril M. Dimitrov, Claudio L. Afonso

**Affiliations:** a National Veterinary Research Institute, Vom, Nigeria; b Exotic and Emerging Avian Viral Disease Research Unit, Southeast Poultry Research Laboratory, U.S. National Poultry Research Laboratory, Agricultural Research Service, U.S. Department of Agriculture, Athens, Georgia, USA; c BASE2BIO, Oshkosh, Wisconsin, USA; d Department of Virology, College of Medicine, University of Ibadan, Ibadan, Nigeria; e Poultry Research Unit, Production Animal Studies Faculty of Veterinary Science, University of Pretoria, Pretoria, South Africa

## Abstract

The first complete genome sequence of a strain of Newcastle disease virus (NDV) of genotype XVII is described here. A velogenic strain (duck/Nigeria/903/KUDU-113/1992) was isolated from an apparently healthy free-roaming domestic duck sampled in Kuru, Nigeria, in 1992. Phylogenetic analysis of the fusion protein gene and complete genome classified the isolate as a member of NDV class II, genotype XVII.

## GENOME ANNOUNCEMENT

Based on serological tests and genome analysis, avian paramyxoviruses (APMVs) have been classified into 13 serotypes designated APMV-1 to APMV-13 (1–4). APMV-1, also known as Newcastle disease virus (NDV), belongs to the genus *Avulavirus*, family *Paramyxoviridae*, order *Mononegavirales*. The virus has a negative-sense, nonsegmented, single-stranded RNA with three genome lengths of 15,186, 15,192, and 15,198 nucleotides (nt) (5, 6). The genome codes for six genes encoding seven proteins: nucleocapsid protein (NP), phosphoprotein (P), matrix protein (M), fusion protein (F), hemagglutinin-neuraminidase protein (HN), large polymerase protein (L), and an additional protein V that is expressed by RNA editing of P mRNA (6). Although these viruses represent a single serotype, antigenic and genetic diversity has been identified among the isolates across the globe (7). Limited genetic information exists for the viruses circulating on the African continent, with only nine genome sequences available in the GenBank database as of 12 December 2015.

A velogenic isolate duck/Nigeria/903/KUDU-113/1992 (hereafter referred to as KUDU-113) (8) was isolated from an apparently healthy free-roaming domestic duck sampled in Kuru, Nigeria, in 1992. The isolate was submitted to the Southeast Poultry Research Laboratory of the USDA in Athens, GA, USA, and further propagated in 9-day-old specific-pathogen-free embryonated chicken eggs. Next-generation sequencing was used to determine the complete genome of the virus. Viral RNA was isolated from the allantoic fluid using a QIAamp RNA viral mini kit (Qiagen, USA). NDV RNA capture and enrichment was performed with three biotin-labeled oligonucleotide probes using Sera-Mag beads (GE Healthcare Life Sciences, USA). Reverse transcription was performed using the Moloney murine leukemia virus reverse transcriptase kit (Thermo Scientific, USA). The cDNA products were purified, tagmented, and amplified into Illumina libraries employing the Nextera XT DNA library preparation kit (Illumina, USA). Fragment size distribution and concentration of the DNA libraries were checked on a Bioanalyzer 2100 using an Agilent high-sensitivity DNA kit (Agilent Technologies, Germany). Paired-end sequencing (2 × 250 bp) of the generated libraries was performed on an Illumina MiSeq instrument using the 500-cycle MiSeq reagent kit version 2 (Illumina). Sequence data were assembled using MIRA version 3.4.0 (9) within a customized workflow on the Galaxy platform (10).

The complete genome length of KUDU-113 is 15,192 nt. It contains six genes in the order 3′-NP-P-M-F-HN-L-5′, with coding sequence lengths of 1,470 nt, 1,188 nt, 1,095 nt, 1,662 nt, 1,716 nt, and 6,615 nt, respectively. Phylogenetic analysis (data not shown) classified the isolate as a member of class II, genotype XVII. The strain KUDU-113 contains three basic amino acid residues between positions 113 and 116 of the fusion protein cleavage site and a phenylalanine at position 117 (_113_RQKR↓F_117_). Such a motif in the deduced amino acid sequence of the cleavage site is specific for virulent NDV isolates (11). Although a genotype XVII genome from West Africa (2008/Mali/ML007/08) was previously published in GenBank (JF966385), this sequence is incomplete and genetic data are missing at both genome termini. Availability of complete genome sequences will facilitate further studies of NDV genetic diversity.

### Nucleotide sequence accession number.

The complete genome sequence of NDV strain duck/Nigeria/903/KUDU-113/1992 has been deposited in GenBank under the accession number KU058680.
